# Dietary Zn—Recent Advances in Studies on Its Bioaccessibility and Bioavailability

**DOI:** 10.3390/molecules30132742

**Published:** 2025-06-25

**Authors:** Joanna Tokarczyk, Wojciech Koch

**Affiliations:** Department of Food and Nutrition, Medical University of Lublin, 4a Chodzki Str., 20-093 Lublin, Poland; joanna.tokarczyk@umlub.pl

**Keywords:** zinc, zinc bioavailability, phytic acid, zinc supplementation, zinc bioaccessibility

## Abstract

Zn is a trace element necessary for the functioning of about 300 enzymes. It plays a biochemical, structural, and regulatory role. It participates in the immune response, proper functioning of the endocrine system, and regulation of gene expression. Its deficiencies are most often caused by the mismatch between dietary intake and the body’s needs. Bioavailability of zinc depends on interactions with other food components. Phytates negatively affect this element’s absorption, whereas proteins, peptides, and amino acids increase its bioavailability. It has been proven that organic forms of zinc are better absorbed than inorganic compounds, like zinc oxide and sulfate. Amino acid combinations with zinc can use amino acid transporters in the absorption process. Estimation of Zn bioavailability and bioaccessibility are based on in vivo and in vitro studies, each having their advantages and disadvantages. The current review aims to gather and summarize recent research on the dietary role of Zn, especially data on bioavailability from food substances promoting/inhibiting absorption, and the latest methods for determining the level of bioavailability of this nutrient.

## 1. Introduction

Zn is a trace element essential to the human body. Its deficiency is a fairly common health problem worldwide, mostly affecting developing countries, where the standard diet is based on food low in Zn and contains large amounts of compounds that disrupt zinc absorption [1,2]. Zinc deficiency is estimated to affect 17–20% of the world’s population [3], most frequently occurring in Asian (19%) and African (24%) countries [4]. Pregnant women, children, the elderly, vegetarians and vegans are the groups most at risk of deficiency. In Africa, zinc deficiency affected 45–83% of newborns and 52–82% of women of reproductive age. Similar data were obtained for these groups in Colombia, Vietnam, and Bangladesh [5]. Inadequate zinc supply is associated with complications during childbirth, premature birth, fetal development disorders, and low birth weight, which in turn result in increased mortality among newborns. Data from a study conducted by Iqbal et al. indicate that in Pakistani pregnant women aged 22–30 years, serum Zn levels were 21.86 ± 7.21 µmol/L, and in non-pregnant women 29.54 ± 7.62 µmol/L [6]. According to the data obtained from a study conducted by Lu et al. among Chinese children aged 6–18 years, zinc deficiency was present in 9.62% of children, with a higher incidence in children aged 6–11 years (10.44%) than in those aged 12–18 years (8.85%). In addition, in the study group, people with anemia, overweight and obesity, rural origin, low economic status, and male gender were predisposed more to zinc deficiency [7].

Zn deficiency significantly contributes to the deterioration of health, and its symptoms are quite nonspecific. Additionally, detecting zinc imbalance in the body is difficult due to the lack of specific biomarkers [8]. Appropriate dietary intake is a key factor to prevent any deficiencies among the population [9]. However, a simple identification of food products rich in this element and an increase in their consumption may not be enough. The key role is to understand the relationship between the bioavailability of Zn and dietary factors which inhibit or promote its absorption. It is known that there are multiple compounds found in food products that enhance or hinder the absorption of zinc [9,10]. Research methods used to determine the bioavailability of various elements, including Zn, are very diverse and include two major categories: in vitro and in vivo techniques [11,12].

This paper aims to present the most recent findings on the role of dietary Zn in health, and especially to discuss dietary factors that affect its bioavailability from various foods. Different chemical forms of zinc salts, which differ significantly in bioavailability, were also discussed. This review also focuses on the bioavailability of zinc from combinations with new compounds, with potential for use as dietary supplements. Particular attention was paid to protein compounds. Data on the bioavailability of zinc from various food groups were also summarized, considering products currently chosen by modern consumers, such as gluten-free and plant-based meat substitutes. The review was based on the results of both in vitro and in vivo studies, the advantages and disadvantages of which were also briefly presented and discussed in the context of studies on Zn bioavailability. Significant differences in the research models described as Zn bioaccessibility and bioavailability studies were also discussed. The review was based mainly on the scientific data from the last 10 years.

## 2. Materials and Methods

**Figure 1:**
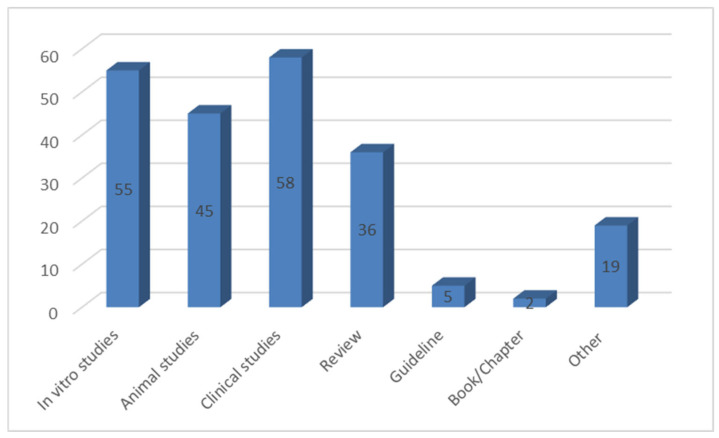
Distribution of study types considered in the review.

To summarize the current knowledge on Zn bioavailability and the factors influencing it, authoritative academic databases such as Scopus, PubMed, and Google Scholar were searched. Keywords used in the database search were zinc, bioavailability, bioaccessibility, factors influencing zinc bioavailability, zinc bioavailability in vivo, zinc bioavailability in vitro, methods for determining bioavailability, phytate and zinc, proteins and zinc bioavailability, different zinc salts, zinc supplementation, zinc deficiency, zinc absorption, Caco-2 cells, digestion model, and in vitro assessment of bioavailability. The review mainly included articles published from January 2015 to December 2024. Results from in vitro, human, and animal studies were included in the review. The publication takes into account dietary guidelines and recommendations developed by governmental and international institutions. The main exclusion criteria were publication date before January 2015, language other than English, and non-peer-reviewed papers. In the case of in vitro studies, the focus was on methods combined with dialysis and Caco-2 cells. In the bioavailability section, review articles were largely excluded, and primarily original papers were included. Figure 1 shows the distribution of study types considered in this review.

## 3. Zn in the Human Body

Zinc is the second most widely distributed trace element in the human body, right after iron [13,14]. Overall, its total content in the human system is around 1.5–4.0 g. All body tissues and body fluids contain a relatively high quantity of Zn: muscles and bones—85% of its total content in the body; skin and liver—11%. Other tissues rich in Zn are the prostate gland and parts of the eye [15,16,17]. Most zinc (>95%) is located intracellularly. The plasma zinc pool is mainly bound to albumin and alpha-2-macroglobulin [18].

**Figure 2:**
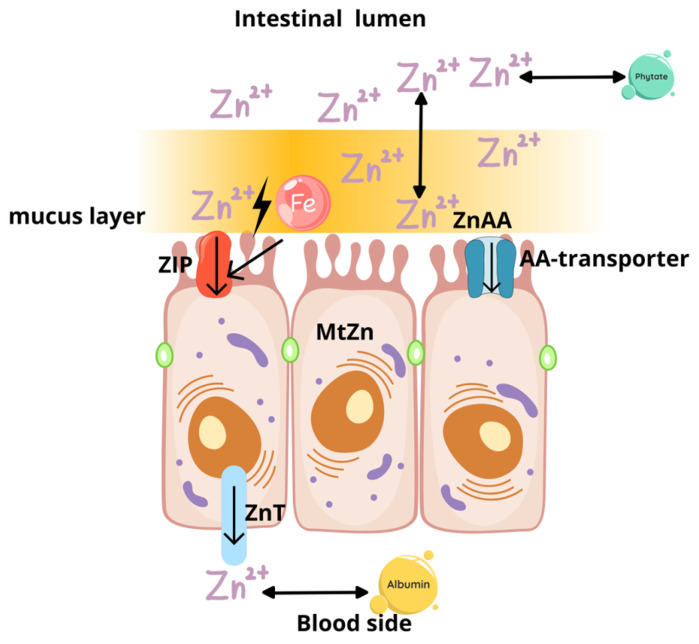
Zinc intestinal absorption.

Zn homeostasis is maintained by the absorption process in the duodenum and proximal jejunum, and the excretion in feces [18]. The essential role in intestinal zinc ion absorption is played by two types of transporters located in different parts of the enterocyte: the Zrt, Irt-like protein family (ZIP) and ZnT transporters, which have opposing roles in maintaining zinc homeostasis. ZnT transports zinc out of the enterocyte into the general circulation or intestinal lumen, whereas ZIP transporters are responsible for transport into the enterocyte [19]. Biochemical aspects of Zn absorption were presented in Figure 2.

*Intestinal absorption of zinc occurs primarily in the duodenum and jejunum. Two main groups of transporters mediate zinc transport: **ZIP** transporters, located on the apical membrane of enterocytes facing the intestinal lumen, facilitate the uptake of Zn^2+^ ions into the cell. **ZnT** transporters, found on the basolateral membrane, export zinc from enterocytes into the systemic circulation, where it binds primarily to albumin and α-2-macroglobulin. Additionally, zinc–amino acid complexes (**ZnAA**) can be absorbed* via *amino acid transporters (AA transporters). Iron competes with zinc for uptake through ZIP transporters, which can affect zinc bioavailability. Intracellular zinc homeostasis is regulated by zinc-binding metallothioneins (**MtZn**), which buffer cellular zinc levels. Moreover, the intestinal mucus layer can bind excess zinc, releasing it when zinc availability is low, thus contributing to zinc homeostasis.*

Zinc is partially excreted in the urine and minor amounts through hair and skin. Zinc absorption depends on the composition of the diet—the amount of other trace elements (especially iron and copper), protein content, phytates, fiber, and citrates [18,20,21]. When the dietary Zn intake is low, fecal and urinary excretion decreases, and intestinal absorption increases to maintain homeostasis [22]. During increased Zn intake, there is chelation of Zn^2+^ by metallothionein in enterocytes and the liver, and increased urinary excretion occur [18]. Intestinal mucins also participate in regulating zinc absorption, as they are capable of buffering excessive amounts of zinc, storing it, and slowly releasing it to enterocytes. Glycoproteins of intestinal mucus, therefore, participate in the absorption of trace elements and prevent their toxic effects [23]. However, proper dietary intake is essential, because adaptive mechanisms are insufficient to effectively eliminate losses of the element [18].

Zn can be found in over 300 enzymes, and is necessary for the proper action of all groups of enzymes: hydrolases, transferases, oxidoreductases, lyases, ligases, and isomerases [24,25]. In the human body, Zn performs a significant function in many biological processes: regulation of gene expression [14], participation in cell apoptosis, transcriptional regulation, and DNA repair [26,27], and therefore may be characterized as having an indirect anti-cancer effect. As a cofactor of superoxide dismutase, it has indirect antioxidant properties. This trace element plays an important role in the cellular and humoral immune response, which is also significant for anti-cancer activity [16,28]. Reduced levels of Zn in the blood were determined in various cancers, e.g., breast, lungs, neck, and head [29,30,31,32].

Zinc is involved in the synthesis of collagen matrix, mineralization, and turnover of bone [33]. Also, the endocrine system requires zinc. This trace element is indispensable for the stabilization of the hexamer structure of insulin, its synthesis, and storage [34,35]. Zn is also involved in the pathway of insulin signaling and promotes lipogenesis and glucose uptake by adipocytes [35]. Zn regulates the synthesis and action of thyroid hormones [36].

Zinc is necessary for reproductive health—it performs a protective function for sperm cells and plays an essential role in spermatogenesis and proper fertilization [37,38]. Zn is also a key component involved in iron metabolism. Studies using intestinal cell cultures have shown that zinc participates in iron uptake and transport by inducing the expression of two iron transporters—ferroportin (FPN1) and divalent metal iron transporter-1 (DMT-1) [39,40,41]. It was also revealed that markers of iron and hemoglobin levels positively correlated with zinc levels in human serum among children [42,43].

In the central nervous system, zinc is one of the most abundant metal ions, where it is crucial in regulating neuronal function and synaptic plasticity. It plays a key role in neurogenesis during embryonic and neonatal development, but also in later periods of life. Brain stroke or neurodegenerative diseases are associated with changes in zinc status. Decreased cognitive functions and impaired learning correlate with deficiencies of this element; however, Zn excess is neurotoxic [44].

### 3.1. Dietary Recommendations for Zn

Insufficiency of Zn induces dysfunction in the human body and results in multifarious diseases. Zinc deficiency is considered prevalent and concerns 17% of the global population [2,18,45,46]. As a major source of this element for the general population is food, the recommended dietary allowance (RDA) established by the US Institute of Medicine is 8 mg/d for women and 11 mg/d for men [47,48]. The latest RDA for Zn for the Polish population has been set at 8 mg/day and 11 mg/day for women and men over 18 years of age, respectively [49]. Increased dietary demand for Zn is observed during puberty, which correlates with the intense growth of bones. Pregnant and lactating women, infants, and children need a larger-than-normal dietary Zn supply [18,50]. The demand for zinc also increases in old age. Intake of Zn in the group aged above 60–65 years averages below 50% of RDA. Deficiencies are aggravated by changes in the absorption of this mineral and drug interactions [47]. In addition, WHO distinguished two RDAs for adults depending on the type of diet: plant-based diets should contain more zinc than meat-based diets, because they contain large amounts of phytates that limit the absorption of Zn [51]. There are disparate recommendations in different countries [52]. The population reference intake (PRI) established by the European Food Safety Authority (EFSA) is 9.4–16.3 mg per day for men and 7.5–12.7 mg per day for women. The daily requirement established by EFSA is based on data on the population’s intake of phytates; therefore, the recommended zinc intake with a diet rich in phytates is higher for both women and men [53]. Guidelines of the German Nutrition Society are different—PRI values are 11–16 mg/d for men and 7–10 mg/d for women [54]. In France, PRI ranges from 9.4 to 14 mg/d and 7.5 to 11 mg/d for men and women, respectively [55]. The highest value of RDA is recommended in India (17 mg/d for men, 13.2 mg/d for women), which is probably due to the high contribution of plant-derived foods in the Indian diet, from which Zn is poorly absorbed [56].

### 3.2. Zn Deficiency and Toxicity

The generality of Zn deficiency is caused by insufficient dietary intake due to low consumption of food abundant in this trace element. The diets of lower-income people based on cheaper foods are meager in bioavailable Zn and rich in components (e.g., phytate) which strangle its intestinal absorption [57,58]. However, it was revealed that wealthy people are subject to the toxicity of this trace element caused by excessive intake of dietary supplements. Food selection has a significant influence on zinc uptake in affluent communities [57]. Table 1 shows the major causes of Zn deficiency. Elderly people, preschool children, and people who exclude animal products from their diet are at high risk of developing zinc deficiency [1].

Vegetarian diets generally exclude products rich in highly bioavailable zinc and are additionally associated with an increased intake of zinc absorption inhibitors such as phytates [62]. According to Chouraqui et al., zinc intake among vegetarian and vegan children is comparable to that of their omnivorous peers. Plasma zinc concentrations are likewise similar across groups, and no clinical signs of zinc deficiency have been observed in either dietary group [63]. In contrast, Neufingerl et al. report that the prevalence of zinc deficiency is highest among vegans, with vegetarians showing similar but slightly higher rates than omnivores [64].Compared to severe Zn deficiency, mild variations in zinc levels are hard to detect because of nonspecific, subclinical symptoms. Additionally, lack of specific biomarkers for Zn levels in the body makes it even more difficult to properly diagnose deficiencies of this element [65]. Plasma zinc levels are not a good indicator of zinc nutrition. Infections, steroid use, contraceptives, pregnancy, and cancer all cause a decrease in the plasma zinc pool. The decrease in plasma zinc levels in these cases is attributed to redistribution to tissues, rather than changes in dietary zinc supply [66]. Inflammation modulates the expression of zinc transporters and metallothionein in various cells. Interleukin 6 regulates the expression of zinc transporters, especially ZIP 14 in hepatocytes, which results in zinc binding to metallothionein in the liver. Zn must be delivered to cells in response to increased protein production, increased reactive oxygen species, and microbial attack [67,68]. Inflammatory cytokines modulate the expression of ZnT and ZIP transporters in both directions, thereby influencing zinc absorption in enterocytes [68].

Research from 2016 showed that the expression of zinc transporters in leukocytes and metallothionein can be alternative biomarkers of zinc status [69]. Knez et al. suggested that the LA:DGLA (linoleic acid:dihomo-γ-linolenic acid) ratio may be helpful for the evaluation of Zn levels. The activity of ∆6-desaturase, which is responsible for the conversion of linolenic acid to dihomo-γ-linolenic acid, is dependent on Zn. Studies on animals and humans have shown that an increased LA to DGLA ratio correlates with zinc deficiency. Interestingly, the LA:DGLA ratio biomarker is able to detect early stages of Zn deficiency. However, the effectiveness of this indicator requires further studies [8]. Regarding limitations of Zn biomarkers, it was concluded that assessment of dietary intake is a good indicator of zinc exposure [53,70].

Acquired deficiency of zinc is marked by skin lesions, impairment of immunity, and diarrhea [71]. Frequently, deep Zn deficiencies are visible in the form of restricted wound repair, disabilities of taste, oligospermia, and hypogonadism [72]. Nakamura et al. proved in animal studies that the skin condition with pressure ulcers worsened due to zinc deficiency [73]. This trace element also participates in vitamin A metabolism and plays a significant role in visual function. Disturbing well-balanced Zn homeostasis may result in night blindness [74]. A deficiency of zinc, a crucial ingredient for endothelial integrity, reduces the protective function of the endothelium and increases the severity of cytokine-dependent inflammatory processes [75]. Since the late 1980s, the relationship between Zn and depressive disorders has been widely studied [76]. Various studies have shown that zinc levels in patients with depression are about 1.85 µmol/L lower than in the control group [77]. Similarly, women with zinc deficiencies have increased symptoms of depression, which was not observed [78,79] in men [80,81]. Similar correlations were observed in rodent studies—a diet enriched with Zn reduced depressive symptoms compared to a diet poor in this element [82,83]. Pharmacological therapy with additional Zn supplementation was proved to be more effective in the treatment of depression than antidepressants alone [84,85]. These data indicate that Zn deficiencies have significant implications for patients with depression.

Zinc also affects the intestinal microbiota. Its deficiencies lead to a deterioration of the diversity of intestinal flora, inflammation, and disorders in gut–brain signaling. The zinc–intestinal microbiota relationship is bidirectional. It is known that microorganisms can produce phytases that release Zn from insoluble complexes with phytates, which increases its bioavailability [86]. The beneficial effect of supplementation with probiotic bacteria on zinc levels has been confirmed by clinical studies [87,88].

*Acrodermatitis enteropathica* is a serious, hereditary disease associated with zinc deficiency [89]. This disease, caused by a mutation in the zinc transporter gene ZIP 4, affects infants during weaning and manifests with dermatitis, diarrhea, and alopecia. In addition, growth disorders, frequent infections, and psoriatic lesions may occur [45].

Zn is a relatively innoxious metal. Toxic effects occur during exposure to high quantities; therefore, acute intoxication is relatively rare. Zn deficiencies are more frequent and have severe, broad implications for well-being and health. Because of its poor bioavailability related to many absorption inhibitors, Zn overload is rarely observed. Long-term supplementation with high doses of zinc is a major trigger of overabundant zinc levels and impairs Cu assimilation [90,91]. Inordinate, chronic Zn ingestion leads to anemia, ataxia, adverse fluctuation in LDL and HDL levels, and immune dysfunction. Abdominal pain, nausea, vomiting, fatigue, and lethargy are signs of acute poisoning [90,92,93]. Tolerable Upper Intake Level (UL) for adults, including pregnant and lactating women, has been set at 25 mg/day [53].

### 3.3. Dietary Sources of Zn

The best dietary sources of zinc are animal-derived foods, especially oysters but also meat (beef, pork, and lamb), fish, and egg products [94,95]. Among the products that contain less zinc than oysters and meat but that are also a good source of this element are milk and dairy products, especially cheese, cow milk, and goat milk, and dairy products differ slightly in zinc content [96]. Legume seeds and nuts (especially cashew nuts) are also a good source of this element; however, their average intake is lower [53,94,95,97,98]. Among cereal products, significant amounts of zinc can be found in buckwheat (32.53–33.76 mg/kg), barley (24.13 mg/kg), and millet groats (23.13 mg/kg). Oat flakes and diet bread made from wheat, rye, and spelt contain large amounts of this element [99]. However, the content of the element in individual products only has a partial impact on supplementing the body’s needs, because the bioavailability of Zn is equally important and has a decisive influence on the absorption of this element from individual products. In general, plant-derived foods are considered inferior sources of dietary Zn, as the absorption of zinc from plant products is limited by fiber, phytates, and oxalates [98]. For breastfed babies, human milk is the source of Zn, but mothers with a mutation of the SLC30A2/ZnT2 transporter gene are marked by poor zinc secretion into the milk [100,101]. Animal products, as already mentioned above, are considered to be better sources of dietary Zn; however, the problem of zinc bioavailability, the influence of individual nutrients, and, last but not least, available and current methods for evaluating its bioavailability from food products are much more complicated issues, which will be described and discussed below.

## 4. Assessment of Trace-Elements Absorption—Differences Between Bioaccessibility and Bioavailability

Foods containing various types of substances (macro- and micronutrients, antinutritional and non-digestible components, and other bioactive constituents) form a complex food matrix, in which all components can interact with each other chemically and physically, influencing the bioavailability of food constituents [102].

The bioavailability of nutrients is defined as a part of the entire intake that is absorbed and used for physiological body functions [103]. There are three phases of bioavailability: intestinal absorption, retention and distribution in the body, and utilization by body cells [104]. The lack of specific biomarkers of Zn utilization is a major problem for studies on its bioavailability. In the case of Fe, isotope tracers cannot be used to determine its bioavailability from whole diets. For this reason in determining the bioavailability of microelements, absorption measurements are used [103]. In these cases, the term bioaccessibility is used, defined as the fraction/amount of a component, released from the food matrix in the gastrointestinal tract, that is capable of absorption. This definition therefore does not include the use of the food component after reaching the general circulation, and therefore [105] does not evaluate the influence of dietary and host-related factors (such as age, gender, and health condition) in other phases of bioavailability [104]. These relationships can only be determined using in vivo models, or better, clinical studies. Moreover, bioaccessibility determined using simulated digestion methods does not provide a full answer to the question of bioavailability and often tends to overestimate its actual value due to the omission of the stages of transport, biodistribution, or excretion of a given micronutrient [106].

Estimation of bioaccessibility/bioavailability of compounds contained in food, including minerals, is possible by using in vitro and in vivo methods [107,108,109,110]. Each of these methods has its advantages and disadvantages, which were described in Table 2. Research about zinc bioavailability is most often carried out using in vitro simulated digestion methods (for these methods, most often the term bioaccessibility is used) [111], and in the case of in vivo methods (bioavailability studies), using stable isotope approaches [112].

### 4.1. In Vitro Methods of Zinc Bioaccessibility Assessment

It would be most beneficial to rely on in vivo models to assess bioavailability, but such studies are expensive, time-consuming, and often burdened with ethical restrictions. As an alternative to these experiments, in vitro digestion models mimicking the gastrointestinal tract were proposed, which are cheaper, easier to perform, and free from ethical issues. Although these models are not fully capable of representing all gastrointestinal conditions, they are often used to predict in vivo digestion outcomes with good results, and can be treated as very effective preliminary studies [106,114].

In vitro digestion is used to determine the bioaccessibility of minerals, trace elements, vitamins, and other bioactive compounds such as polyphenols [106]. The terms bioaccessibility and bioavailability are used interchangeably in many publications on Zn, including those based on in vitro methods. The concept of bioavailability refers not only to absorption but also to the distribution and use of the component by the body, which can only be determined in vivo. In the case of zinc, the lack of specific biomarkers makes it difficult to estimate its bioavailability. However, in the latest publications, authors using in vitro methods use the term bioaccessibility to refer to the release of zinc from the food matrix/pharmaceutical preparation and the degree of its absorption. In this review, in vitro methods were defined as bioaccessibility studies and in vivo studies were assigned to determine bioavailability. According to the authors, this division is unambiguous. However, the original phrases in accordance with the scientific source have been retained, even if, in the authors’ opinion, the terms bioaccessibility/bioavailability were used incorrectly.

One of the first models to reproduce gastrointestinal digestion was the model proposed in 1981 by Miller et al. [115]. This model was based on the reproduction of the conditions of the gastrointestinal tract lumen—temperature, pH, mixing, and enzyme composition. Until now, it has been widely used with some modifications in Zn bioaccessibility assessment [110,116]. Static digestion models are based on constantly stirred and thermostatic systems, in which the appropriate pH is set before a specific digestion stage [102]. For this digestion method, the INFOGEST protocol was developed, describing a set of digestion parameters (pH, ionic composition, enzyme activity, endogenous surfactants) [117]. In reality, the full reproduction of in vivo conditions is not easy and almost impossible, and, therefore, such a simplified approach of static models will never fully simulate the constantly changing biochemical and physical conditions of the human gastrointestinal tract. For this reason, dynamic models have been developed. Although they are more expensive and require more work, they have a higher potential of replacing clinical studies [102,118]. Dynamic models attempt to reflect parameters such as the actual shape of the stomach, peristaltic movements, and biochemical parameters changing over time [113]. However, static digestion models are still most often used in Zn bioaccessibility studies [110,111,116,117].

The in vitro models used are intended to digest the food matrix, but their use without combining with methods simulating the absorption of the element would be unjustified in the context of bioaccessibility testing. Dissolved nutrients released from the food matrix can be absorbed in the small intestine. In the final stage of digestion, several methods are employed to investigate zinc absorption. One of them measures the soluble fraction remaining after digestion in the supernatant, which is then theoretically ready to be absorbed in the small intestine [12,119,120,121]. The uptake of zinc can also be studied using biological membranes such as animal intestinal sections or epithelial cell lines [122]. Their use includes all types of membrane transport. However, during the preparation of animal membranes, key transport proteins may be destroyed. Cell culture models are more advantageous, especially Caco-2 cells isolated from human colon adenocarcinoma [113].

Caco-2 cells are easy to culture, form a layer of cells similar to the intestinal epithelium, and contain some digestive enzymes and membrane transporters [113,123,124]. Caco-2 cells have been used to elucidate and understand the mechanisms of zinc transport and absorption [125]. They are currently most effectively used in conjunction with in vitro digestion models to determine Zn bioaccessibility from foods [126] and dietary supplements [127,128]. They are also used to investigate the effects of potential new compounds that may enhance zinc availability [122,129,130]. It has been suggested that intestinal mucins participate in zinc resorption; therefore, it may be beneficial to use co-cultures of mucin-producing Caco-2/HT-29-MTX cells in studies on intestinal zinc absorption [125,131]. Sauer et al. additionally studied the uptake of zinc from amino acid complexes using enterocytes differentiated from human induced pluripotent stem cells (hiPSC) derived from a healthy volunteer and a patient with AE [10].

Semipermeable dialysis membranes with specific pore sizes that mimic the small intestine are also successfully used in the assessment of zinc bioaccessibility [110,111,116,132,133,134]. Dialysis results have been shown to correlate with human absorption data [135]. However, pore size must be considered when selecting semipermeable membranes. Mostly membranes with pore sizes ranging from 10 to 14 kDa are used [110,111,116,133]. Gomez et al. studied zinc availability from milk formula. They have evaluated gastrointestinal extracts from the digested milk formula using SEC-ICP-MS (size-exclusion chromatography coupled to inductively coupled plasma-mass spectrometry) analysis. They showed that Zn was bound to molecules ranging from 1 to 7 kDa, but the amount of the dialyzed zinc (1–10%) was lower than expected (dialysis membranes with a molecular weight cutoff of 10–12 kDa were used). Although zinc was available for absorption, it was not completely dialyzed. The study by Gomez et al. showed that the results obtained from in vitro dialysis should be interpreted with caution, taking into account the many factors influencing this method and, at best, verified by SEC-ICP-MS speciation analysis or in vitro models using Caco-2 cells [136]. Moreover, artificial intestinal membranes are only able to simulate the passive transport of substances, not other forms of transport [113].

The choice of the appropriate method of simulating absorption is important in the case of zinc, because its absorption occurs in two ways. Active transport occurs with the participation of two types of transporters, ZIP proteins and ZnT proteins. This transport dominates at low zinc concentrations, while at higher concentrations it is absorbed by passive diffusion [9].

To determine the amount of zinc absorbed and to estimate its bioaccessibility, atomic absorption spectrometry (AAS) [116], inductively coupled plasma-mass spectrometry (ICP-MS) [111], and inductively coupled plasma-emission spectrometry (ICP-OES) [126] are used.

### 4.2. In Vivo Methods of Zn Bioavailability Assessment

In vivo models for determining the bioavailability of zinc involve studies using animal (rats, dogs, pigs, broilers) models and clinical studies. In vivo and clinical studies allow for precise assessment of Zn bioavailability and best reflect the complex relationships in the lumen of the human digestive tract, considering the mechanisms of passive and active absorption of Zn. They enable the determination of parameters such as the maximum concentration in plasma (Cmax), the time to reach the maximum concentration in plasma, and the area under the curve (AUC), which helps in assessing bioavailability [113].

#### 4.2.1. Studies Performed in Animal Models

Due to its expensive nature, animal studies on Zn bioavailability are less commonly performed than in vitro studies [137]. The first animal models used to assess zinc bioavailability were rat models. Body weight gain, tissue zinc content, and zinc absorption were assessed using isotope techniques, and zinc excretion was monitored. However, because these animals can produce phytases, they are not good models for assessing zinc bioavailability, especially in the case of diets containing phytates [135]. Phytase activity is low in rat pups [135], which is why they have been preferred by some researchers [138].

Currently, other animal models are used to study zinc bioavailability. For example, Kang et al. studied the potential of using Zn- and Se-enriched *Lactobacillus plantarum strains* as a dietary supplement with improved zinc and selenium bioavailability in mouse models [139]. Similarly, Sauer et al., in addition to in vitro models, also used mouse models to estimate the bioavailability of zinc amino acid complexes [10]. The influence of chemical form on zinc bioavailability was studied by Chen et al. in broilers [140].

Tako et al. evaluated the bioavailability of zinc methionine using chicken embryos (Gallus gallus). After intra-amniotic administration of zinc methionine, they assessed the functionality of the small intestine and the expression of zinc transporter mRNA. An increase in the area of intestinal villi was demonstrated after 96 h of zinc methionine administration, and after 48 h, there was a 200% increase in the expression of the Znt1 transporter compared to the control group. This study initiated the use of the Gallus gallus model in the assessment of zinc bioavailability [141]. Gallus gallus was also used to assess the usefulness of a potential biomarker of zinc deficiency, which is the LA: DLGA ratio [142]. A well-known phenotype, rapid maturation, similarity of the intestinal microbiota, and, above all, a high level of homology (approximately 73%) of trace element transporters in broilers and humans are the advantages of the Gallus gallus model [143,144].

In the case of animal studies, they require the selection of an appropriate research model, because the tissues of some animals (e.g., adult rats) may be characterized by different mechanisms of Zn absorption, and therefore may not constitute an appropriate model for the human body, and the obtained results may be false. In vivo methods also do not allow for rapid changes in the tested compounds or nutritional mixtures, whereas in vitro methods have a great advantage, allowing for quick and cheap tracking of changes in bioavailability under the influence of numerous external factors.

#### 4.2.2. Clinical Studies

Clinical studies utilize safe, stable isotope-labeled tracers, whose absorption, distribution, metabolism, and excretion closely resemble those of the unlabeled compound. To evaluate the bioavailability of Zn, the test subject consumes a meal labeled with such an isotope and also receives a second isotope intravenously (for which absorption is assumed to be 100%). Subsequently, the ratio of the two isotopes in urine or plasma is analyzed, allowing for the determination of the total absorption of zinc from the meal and the fractional absorption of the element [112]. Methods employing stable isotopes are utilized in zinc bioavailability studies that include children and adults, with meals or products administered to the subjects primarily labeled with the isotopes ^67^Zn and ^70^Zn [145,146,147,148].

**Figure 3:**
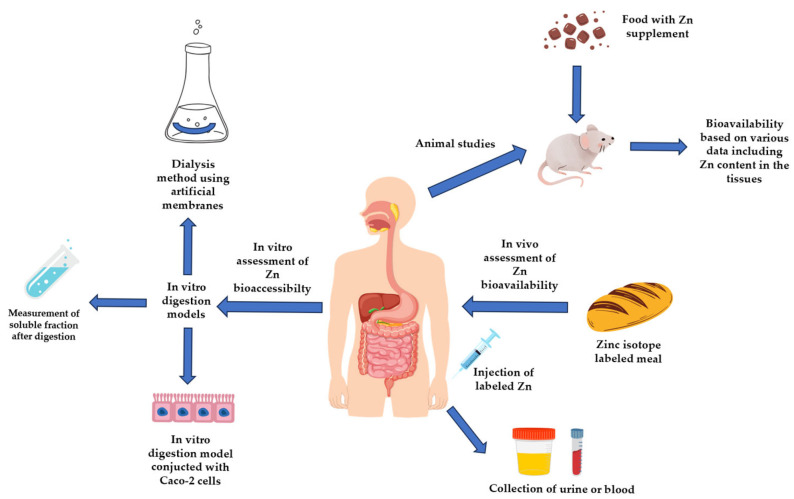
Summary of methods used to estimate Zn bioaccessibility/bioavailability.

Studies on humans are the only experiments in which the exact amount of Zn that enters the bloodstream can be determined. However, these methods are expensive, time-consuming, and require appropriate bioethical consent. Figure 3 summarizes methods used in the estimation of bioaccessibility and bioavailability of Zn.

## 5. Bioaccessibility/Bioavailability of Zinc

The main site of Zn absorption is primarily the duodenum and jejunum [125]. Zinc absorption depends largely on its dietary intake. It is assumed that about 1/3 of the consumed element is absorbed. Zinc is absorbed by about 60–70% when taken with a liquid meal on an empty stomach [9]. However, its absorption from a normal, mixed diet ranges from 16% to 50% [125]. The amount of zinc contained in the food itself plays a limiting role in further absorption, which is related to the saturation of transporters of this trace element [10,60]. Active transport through ZIP and ZnT proteins dominates at low zinc concentrations in the intestinal lumen. However, at higher zinc concentrations in the intestinal lumen, zinc is transported by passive diffusion [9,125]. According to kinetic studies, passive zinc transport occurs at zinc intakes above 30 mg/d [149].

Various food ingredients have a crucial impact on the absorption process. Zn is released from food during digestion and absorbed in an ionized form. However, the released zinc ions can form compounds with ligands such as phytates, organic acids, or amino acids, which results in variable solubility and improves or deteriorates the absorption [9]. The crucial factor for zinc absorption is its dietary origin: plant-based foods contain less bioavailable Zn than meat [45]. Using an in vitro digestion model with Caco-2 cells, Latunde-Dada demonstrated significantly higher absorption of Zn from beef burgers than from their plant-based substitutes [126]. As reported by Hunt et al., Zn was absorbed much less from a lactoovovegetarian (26%) than from a nonvegetarian diet (33%) [150]. Meat is a good source of proteins, vitamins and minerals. Menezes et al. studied the bioaccessibility of zinc and other elements from different types of meat after heat treatment using in vitro digestion methods. The highest Zn bioaccessibility was estimated for boiled pork (20%) and beef, which was grilled and baked at 180 °C for 45 min (20%). In comparison, the bioaccessibility of Zn from boiled and grilled chicken was 15% and 12%, respectively. The lowest results were observed for roasted (180 °C, 60 min) beef (12%), followed by chicken (8%), and roasted (180 °C, 45 min) and microwaved pork (10%) [151]. Amiard et al. studied the bioaccessibility of trace, essential, and toxic metals in different species of shellfish from Western Europe and Asia. The bioaccessibility of Zn from these products was on average 65%, the highest was observed for the oyster *C. gigas* (82%) and the lowest from the mussels (34%) [152]. According to Melse-Boonstra, the bioavailability of zinc from milk and dairy products ranged from 25 to 30% [153]. It was revealed that the bioavailability of Zn from human milk is much higher (41 ± 9%) in comparison to cow’s milk (28 ± 15%), and the soy protein isolate formula (14 ± 4%) [154]. Moskwa et al. studied the bioaccessibility of Zn from different types of nuts. The highest results were estimated for pistachios (48.10 ± 12.71%), macadamia nuts (39.44 ± 7.87%), and pecan nuts (16.96 ± 3.33%), whereas the lowest were for Brazilian nuts (3.01 ± 1.10%) and peanuts (5.23 ± 2.38%). Walnuts were characterized by a moderate Zn bioaccessibility at the level of 10.73 ± 4.31% [110]. This study showed that even within one group of food, Zn bioaccessibility may vary significantly between particular products. Rogaska et al. determined the bioavailability of zinc from gluten-free breads using an in vitro digestion method combined with measurement of the soluble fraction. The bioavailability of Zn from buckwheat bread was twice as high as from rice bread. [155]. Previous studies conducted by Regula et al. also estimated higher bioavailability of zinc from buckwheat bread (51.7 ± 1.33%) than from rice bread (36.6 ± 2.31%). Bioavailability of this element from buckwheat bread with added milk was lower (34.5 ± 4.04%) than from rice bread with added milk (53.1 ± 4.24%). On the other hand, the addition of milk and seeds to both types of bread increased bioavailability for rice and buckwheat bread, resulting in 69.8 ± 0.19% and 74.8 ± 2.04%, respectively [156].

In order to reduce dietary Zn deficiency, strategies have been developed to enrich and biofortify food with Zn, especially cereals and legumes, which are the basis of the diet in populations with a high risk of Zn deficiency [157]. As shown by animal studies (Gallus gallus model) by Knez et al., higher amounts of Zn in biofortified wheat correlated with higher Zn absorption [158]. Studies by Gomes et al. using the Gallus gallus embryo model showed that zinc-biofortified cowpea (*Vigna unguiculata* L. Walp.) flour improves brush border function, increases the expression of genes encoding Zn and Fe transporters, and modulates beneficial gut microbiota [159].

As was already mentioned above, various food components have a strong impact on the bioavailability of Zn, therefore the following sections will discuss several nutrients and non-nutrients that significantly influence the bioavailability of this trace element. The bioavailability and absorption of this element from different chemical forms will also be discussed.

### 5.1. The Influence of Phytates and Dietary Fiber

#### 5.1.1. The Influence of Phytates on the Bioavailability of Zn

Phytic acid and its derivatives, often called phytates, are non-nutritive components of food products, present in significant amounts in cereals and legumes. They reduce the bioavailability of various mineral compounds by forming poorly absorbable complexes. In developing countries, meals are based mainly on cereal products, which may result in micronutrient deficiencies (including Fe, Zn, and vitamin A) [160].

The key aspect is not the presence of phytates in the diet itself but the molar ratio of phytates to zinc. In the studies conducted by Fredlund et al., the subjects were given wheat rolls labeled with ^65^Zn and ^47^Ca radioisotopes with and without various levels of phytates. The results were obtained after measuring the retention of radioisotopes in the whole body. At a molar ratio of phytate to zinc of 5–15, Zn absorption dropped from 21% to 11–16%, while at a molar ratio above 15, zinc absorption dropped below 11% [161]. In the studies by Hambidge et al., which included pregnant women (8th and 34th week of pregnancy) and breastfeeding women (2nd and 6th month of lactation), the results obtained confirmed previously mentioned conclusions. The control group received corn with a phytate content of 710 mg/100 g of dry matter, and the study group received corn with a reduced phytate content (160 mg/100 g of dry matter). The amount of completely absorbed zinc (TAZ) was higher at a lower molar ratio of phytate to zinc. It was also observed that in both groups, TAZ increased in the 3rd trimester of pregnancy compared to the first trimester, it was the greatest in the 2nd month of lactation, and decreased again in the 6th month of lactation. This indicates that the mechanisms of zinc homeostasis allow for partial compliance with the increased demand for this nutrient [162].

Numerous other experiments have also shown that phytates reduce the bioavailability of minerals [163,164]. In two independent studies, the first by Mayer-Labba et al. and the second by Latunde-Dada et al., the bioavailability of minerals—zinc and iron from plant-based meat substitutes—was investigated [126,165]. Mayer-Labba et al. estimated the bioavailability of minerals from meat substitutes based on the determination of the molar ratio of phytate to zinc/iron. Mineral contents were determined by atomic absorption spectrometry (AAS) and phytate by high-performance ion chromatography (HPIC) [165]. The experiments conducted by Latunde-Dada et al. included the assessment of the bioavailability of minerals, including zinc, from meat and plant burgers in in vitro digestion models using Caco-2 cells [126]. It is clear from both experiments that plant-based meat substitutes were characterized by lower Zn bioavailability than meat products. It was also shown that only mycoprotein substitutes characterized by low phytate content were a good source of readily bioavailable zinc [126,165].

The effect of Ca on Zn bioavailability in the presence of phytates is not clear. From the results of animal studies, it can be concluded that calcium in the presence of phytates reduces zinc absorption [166,167]. Mendoza et al. showed that higher dietary calcium content correlated with lower zinc absorption from a supplement that had no phytate content but consisted of milk powder, a trace element mixture (iron sulfate and zinc sulfate), and components rich in phytic acid—cereal and legume flours [168]. However, Hunt et al. in their experiment with 10 women confirmed the negative effect of phytates on zinc bioavailability, but without a significant effect of calcium [169]. Miller et al., using a mathematical model based on the analysis of secondary data and biochemical studies, observed a visible, although small, effect of calcium on increasing zinc absorption in the presence of phytates [170].

Milk—one of the richest sources of calcium in the diet—increased the bioavailability of zinc from rice with a high amount of phytates. The absorption fraction of zinc for the mixture of milk and rice (741 mg calcium, 297 mg phytates) was 20.8%, while for the mixture of water and rice (33 mg calcium, 297 mg phytates), it dropped to 12.8%. However, the highest bioavailability of zinc in the absence of phytates in the meal was observed for water (72.3% for water, 27.8% for raw milk, 25.5% for UHT milk, and 19.7% for the mixture of milk and water). It is likely that other components of milk, such as proteins, eliminated the limiting effect of calcium on Zn absorption [147]. The conclusions of Talsma et al. regarding the beneficial effect of milk on zinc bioavailability from phytate-rich meals are consistent with the results obtained in other studies [171]. It is likely that citrate, the main zinc-binding ligand in milk, positively affects the bioavailability of this element from dairy products and milk. Zinc is better absorbed from human milk than from cow’s milk, which may correlate with the higher concentration of the zinc–citrate complex in human milk [125].

Although phytates are defined as antinutritional compounds that inhibit the absorption of some minerals, in recent years, their beneficial properties for health have been discovered. According to Grases et al., phytate and its hydrolysates inhibit the crystallization of calcium salts [172]. Animal studies by Grases et al. have shown that long-term consumption of a large dose of sodium phytate does not affect the bioavailability of Ca, Mg, Fe, and Mn. However, in the case of zinc, a decrease in bioavailability was observed [173].

The effect of phytates on Zn bioavailability can be reduced by the enzymatic degradation of these compounds. Phytates, or in chemical terms, hexaphosphates and pentaphosphates, limit zinc absorption, whereas derivatives with lower molecular masses, tetra and triphosphates, have little effect on Zn absorption. The decomposition of higher phytate derivatives to tetra and triphosphates occurs with the participation of the enzyme phytase, which, in negligible amounts, is present in the human small intestine. Moreover, the level of phytate degradation in the diet also depends on the activity of plant and bacterial phytases. The plant-derived enzyme is activated during fermentation or heat treatment of food products, which improves Zn absorption [125]. Ahmed et al. showed that with increasing fermentation time (0–72 h), the amount of antinutritional compounds (phytates, tannins, and oxalic acid) decreased in kisra bread made from Koreeb (Dactyloctenium aegyptium) seeds, which contributed to increased bioavailability of Zn, Fe, and Ca. Fermentation for 18, 36, 54, and 72 h, respectively, resulted in phytate reduction by 4.9, 19.7, 26.9, and 26.3%, and increased zinc bioavailability by 50, 54, 59, and 63% compared to unfermented grains (46%) [174]. Heat treatment without soaking biofortified beans reduces the amount of phytates (from 2301.0 mg/100 g for raw beans to 1803.23 mg/100 g and 1813.90 mg/100 g for cooking, oven-drying and cooking, and freeze-drying beans, respectively). Soaking beans before heat treatment further reduces the amount of phytates (a decrease to 1530.30 mg/100 for cooking, freeze-drying, and 1933.07 mg/100 g for cooking, oven-drying). A significant effect of soaking beans before heat treatment is visible. Despite the decrease in the amount of phytates in beans, there is a loss of minerals as a result of soaking and cooking. This is explained by the fact that zinc absorption by Caco-2 cells from raw beans was higher (36.26 µg) than for beans after treatment (24.68–31.25 µg). Considering the fact that beans are cooked before consumption, the combination with oven drying with or without soaking seems to be the best way to optimize Zn bioavailability [156]. In a double-blind, randomized study by Zyba et al., Gambian children aged 18–23 months were given porridge with SQ-LNS (small-quantity lipid-based nutrient supplement) with or without fortification with endogenous phytase. The FAZ (fractional absorption of zinc) increased from 8.6% to 16% in the phytase-enriched meal. The TAZ (total absorption of zinc) from the phytase-enriched meal was more than twice that from the unenriched meal [175]. The beneficial effect of phytase addition to meals rich in phytates on Zn bioavailability was also confirmed by other authors [176,177].

Intestinal microbiota degrades phytates. Mitsuokella jalaludinii, common in human microbiota, degrades phytates. In cooperation with Anaerostipes rhamnosivorans, it converts these compounds into short-chain fatty acids. It improves the absorption of minerals. Additionally, it increases the integrity of the intestinal barrier, as demonstrated on Caco-2 cells incubated with co-culture supernatants of these microorganisms [178]. The commensal Escherichia coli also produces phytase [179]. While diets rich in phytates reduce zinc bioavailability, they may simultaneously promote the growth of probiotic microorganisms [180,181]. As previously discussed, the relationship between zinc and the gut microbiota is bidirectional: zinc deficiency has been associated with reduced microbial diversity [180].

#### 5.1.2. The Influence of Dietary Fiber on the Bioavailability of Zn

Foods high in fiber, including whole grains, unrefined cereals, and legumes, contain high levels of phytates. Many populations rely on these products as the foundation of their diets. The effect of dietary fiber on the bioavailability of nutrients is related to the presence of phytates. A study by Foster et al. demonstrated that higher dietary fiber intake is associated with increased phytate consumption and a greater risk of zinc deficiency in both healthy women and those with type 2 diabetes [182]. Dietary fiber may reduce the absorption of nutrients by thickening the chyme in the intestine and thereby impeding contact of nutrients with the surface of enterocytes [183].

The consumption of high-fiber foods is encouraged, as they promote intestinal microbiota diversity. Enriching meals based on plant products with phytases can significantly increase the absorption of Zn from the diet. This in turn can contribute to the reduction of the Zn deficiency in underdeveloped countries, where the basis of the diet are plant products, from which zinc is most often much less absorbable compared to meat products.

### 5.2. The Influence of Proteins and Peptides

#### 5.2.1. The Influence of Proteins on the Bioavailability of Zn

Protein components of food are considered to enhance zinc absorption. Animal proteins, in particular, are responsible for this effect—when added to a plant-based meal, they increase the bioavailability of this element [125,129,147]. Protein digestion products—peptides and amino acids—form more easily absorbed complexes with Zn, as they utilize amino acid transporters during absorption [10].

Christensen et al. described the effect of whey protein osteopontin (OPN) on the ability to bind zinc ions. The bioavailability of zinc from the OPN-Zn complex was studied using in vitro model with Caco-2 cells. The OPN-Zn complex enhanced zinc absorption in the presence of phytic acid compared to inorganic zinc salts, suggesting its potential as a functional food additive [129]. The bioavailability of Zn from dietary supplements containing two protein-rich species of cyanobacteria, *Arthrospira platensis* and *Arthrospira maxima* (commonly known as Spirulina), was found to be higher than that fromZn gluconate [184].

Milk proteins are carriers of many bioactive compounds, including minerals. The ratio of casein to whey protein is important for the bioavailability of zinc from milk, which is why this element is better absorbed from human milk (casein to whey protein ratio 40:60) than from cow’s milk (casein to whey protein ratio 80:20) [9]. Shilpashree and colleagues studied the bioavailability of a mineral complex with succinylated sodium caseinate in in vitro digestion models using Caco-2 cells. The mineral components (Zn and Fe) showed higher bioavailability from such a complex than from the inorganic form. Also, the amount of zinc transported, retained, and taken up in Caco-2 cells was higher from the unmodified protein–mineral complex than from the inorganic compounds [130].

#### 5.2.2. The Influence of Peptides on the Bioavailability of Zn

Complexes of peptides derived from oysters (*Crassostrea gigas*) with Zn were characterized by better zinc bioavailability in the presence of phytates compared to ZnSO_4_. Zinc from these complexes is absorbed by two routes: through the zinc ion channel and small peptide transport [185]. Wang et al. studied the effect of a zinc complex with soybean meal hydrolysate on the absorption of this microelement in mouse models. The combination of high solubility and small particle size had a beneficial effect on zinc levels and the activity of zinc-dependent enzymes in mice. It was also found that this complex, despite having a lower Zn ion binding capacity than the peptide obtained from the hydrolysate used, had a similar effect on zinc absorption [186,187].

Casein phosphopeptides (CPP) are bioactive peptides derived from casein hydrolysis, which have mineral-binding sites. They therefore play an important role in mineral absorption [188]. Casein phosphopeptides increase bioavailable zinc from diets high in phytates, which suggests that such a protein can effectively reduce the negative impact of phytates on the bioavailability of this element [122].

Soluble chelate complexes form with divalent metals, including zinc, and peptides containing amino acid residues such as cysteine, histidine, aspartate, glutamate, and serine. The stability of these complexes during digestion and the structure–function relationship are of key importance for nutrition and health. Therefore, natural zinc-chelating food peptides may have the potential as ingredients of functional foods, nutraceuticals, or dietary supplements, which may increase Zn bioavailability from various foods [189].

### 5.3. The Influence of Other Elements

Zinc absorption may be affected by large doses of iron; however, available scientific data are inconclusive. Simultaneous supplementation of iron and zinc on an empty stomach results in limited zinc absorption, with this effect being observed at a dose of Fe above 25 mg. However, this effect is not observed when both microelements are supplemented during a meal. Fe fortification of food also does not affect Zn absorption [170,190].

The effect of iron on zinc bioavailability can be explained by competition for transporters. The ZIP (ZRT/IRT-like Protein) family of transporters is primarily responsible for Zn^2+^ uptake; however, several members also facilitate the transport of other divalent cations, including Mn^2+^ and Cd^2+^. Notably, ZIP14 and ZIP8 have been shown to mediate Fe^2+^ transport, particularly under conditions of elevated iron concentrations. ZIP14, in particular, plays a role in maintaining iron homeostasis by transporting non-transferrin-bound iron (NTBI) [191]. Although earlier studies suggested that zinc might interfere with iron absorption through the divalent metal transporter 1 (DMT1) [192], it is now well established that Zn^2+^ is not a substrate for DMT1. Therefore, the interaction between zinc and iron during absorption is primarily attributed to their competition for ZIP family transporters [193].

As observed by Esamai et al., iron fortification of corn given to 6-month-old infants did not significantly affect zinc absorption. The total amount of zinc absorbed in the group receiving food fortified with both elements was 0.85 mg/day, while in the group without iron supplementation it was 0.72 mg/day. Both values were higher than in the control group (0.24 mg/day) [194]. Also taking an iron preparation (100 mg Fe/day) did not affect zinc metabolism in pregnant women, as the FZA (fractional zinc absorption) increased with the development of pregnancy regardless of supplementation. The internal mechanisms of the body to maintain the homeostasis of this element are much more important than external factors such as the dose of zinc, its form, or the presence of substances promoting or inhibiting its absorption [195]. However, more recent data from a clinical trial conducted in a large group of women of reproductive age (760 women) indicate that iron supplementation at a dose of 60 mg/day may interfere with zinc absorption and metabolism [196]. Similarly, Troost and colleagues in an earlier study observed a negative, dose-independent effect of iron supplementation on zinc absorption [197].

The aforementioned scientific data show that the effect of Fe on limiting Zn bioavailability is ambiguous. It probably is heavily dependant on the iron dose, as well as the adopted research model, including the study group. Perhaps factors such as age, health, or physical condition of the studied population are important for the discussed interaction. More studies are definitely needed to fully understand this relationship.

Zhang et al. studied the bioaccessibility of zinc, selenium, and cadmium from rice in the presence of dietary supplements containing vitamins, coenzyme Q10, procyanidins, copper, and methionine in simulated in vitro digestion models. They showed that the bioavailability of zinc increased in the presence of vitamin C, procyanidins, and coenzyme Q10. On the other hand, the bioaccessibility of Zn decreased in the presence of vitamin E, B_6,_ and copper, respectively [121].

### 5.4. Bioaccessibility/Bioavailability Depending on Different Chemical Forms of Zn

One of the options for compensating for zinc deficiency, in addition to a proper diet, is supplementation. There are many dietary supplements on the market that differ in the chemical form of zinc. The most common forms found in dietary supplements and pharmaceutical preparations are gluconate, glycinate, picolinate, sulfate, and zinc oxide. These forms differ not only in solubility or sensory properties but also in the bioavailability of zinc [190].

#### 5.4.1. In Vitro Studies

Studies involving humans are complicated, very expensive, require a lot of time and the approval of an appropriate bioethical commission, and recently also special insurance of the participants. Therefore, in vitro experiments on the bioaccessibility of various nutrients, especially minerals, are currently popular and frequently performed.

Ośko et al. determined the bioaccessibility of zinc from dietary supplements using an in vitro digestion model. Various chemical forms, pharmaceutical forms, and doses were taken into account. Based on the conducted experiment, it was found that zinc sulfate has the lowest bioavailability (1.13%), while a dietary supplement with zinc bis-glycinate has the highest (9.38%). The bioaccessibility of zinc citrate (1.99%) was the lowest among the organic salts tested. The bioaccessibility determined for zinc methionine and picolinate was 3.38% and 3.15%, respectively. In this study, the influence of dietary factors was not taken into account—only the dietary supplements themselves were used. Comparing the absorption of zinc from gluconate, the bioavailability was higher for capsules (6.19%) than for tablets (4.48%) [116].

The previous section also describes the bioaccessibility of zinc from complexes with proteins and peptides. Briefly, the compounds have the potential to supplement zinc during dietary deficiencies, due to their greater bioavailability than inorganic forms. Conjugates of zinc with amino acids may have an advantage over classical zinc compounds in dietary supplements. Sauer et al. demonstrated that in cells from a patient with AE (*Acrodermatitis enteropathica*), absorption of zinc conjugates with amino acids was not impaired, in contrast to absorption of ZnCl_2_. These conjugates used amino acid transporters. This is particularly important for patients with congenital mutations of genes encoding zinc transporters (ZIP family transporters) [10]. Similarly, Zhang et al. showed that zinc pentapeptide-chelate from sweet almond pomace had higher solubility and zinc transport capacity than zinc gluconate and sulfate, making it a potential ingredient in dietary supplements. The same study also showed better absorption of zinc gluconate than sulfate [198]. Dietary supplements containing *Chlorella vulgaris* extracts are popular due to their nutritional value and beneficial effects. However, Muszyńska et al. determined that the bioaccessibility of Zn from these supplements was negligible, which does not make them good sources of this element. The zinc content in the residue after the 90 min gastric phase ranged from 0.42 ± 0.004 to 1.99 ± 0.013 µg/g preparation, and after the 90 min intestinal phase ranged from 0.05 ± 0.002 to 0.67 ± 0.003 µg/g preparation, depending on the dietary supplement [199].

#### 5.4.2. Animal Studies

According to Chen et al., in broilers, the bioavailability of zinc from complexes with glycine or methionine is greater than that of zinc sulfate [140]. Better bioavailability of zinc from the glycine complex compared to zinc sulfate in young lambs was demonstrated by Deters et al. [200]. The methionine complex had an advantage over the compound with glycine [140]. Farhadi Javid et al. in a study on broiler chickens showed that the relative bioavailability of zinc chelate with threonine to zinc sulfate, based on zinc content in tibia bone, was 117.65%. The relative bioavailability of zinc threonine to zinc sulfate based on tibia bone ash content, body weight, feed conversion ratio, superoxide dismutase enzyme, and LDL and HDL content was 156.46, 418.75, 173.91, 193.45, 159.43, and 278.63%, respectively [201]. Yu et al. concluded that basic zinc chloride showed better bioavailability than zinc sulfate in broilers. The relative bioavailability of zinc chloride to zinc sulfate based on body weight gain was 110.82%, whereas that based on zinc content in pistil bone, CuZn superoxide dismutase (CuZn-SOD) activity, plasma alkaline phosphatase (ALP), and divalent metal ion transporter (DMT1) mRNA expression ranged from 108 to 119% [202]. According to Hu et al., zinc–protein complexes show higher bioavailability compared to zinc sulfate, which is associated with the promotion of the expression of ZIP3, ZIP5, and y+LAT2 transporters in the duodenum of broilers [203].

#### 5.4.3. Human Studies

A randomized, double-blind study by Rosado et al. on Zn absorption from corn tortillas fortified with zinc oxide or zinc sulfate showed that both chemical forms had similar zinc absorption as measured by the stable isotope method. The same study found that the effect of iron supplementation (30 and 60 mg/kg corn flour) on zinc bioavailability was negligible [204]. Also, an earlier experiment conducted by Lopez de Romana et al. showed that zinc oxide and zinc sulfate did not differ in the amount of absorbed element [205]. Studies on the bioavailability of various chemical forms of Zn have shown the advantage of organic forms over zinc oxide [21,206,207]. Wegmüller and colleagues compared zinc absorption from dietary supplements containing citrate, gluconate, and zinc oxide. The study was conducted with the participation of 15 healthy adults who received a supplement containing 10 mg of zinc regardless of the meal. Zinc absorption from citrate was similar to that from gluconate (for citrate 61.3%, for gluconate 60.9%) and was higher than from zinc oxide (40.9%) [21].

Di Silvestro et al. compared the effects of supplementation with two chemical forms of zinc, glycinate, and gluconate, on plasma zinc concentrations. Women aged 18–24 years were given supplements of either zinc salt or a placebo with meals for 6 weeks. Blood samples were taken before and after supplementation. Plasma zinc status was found to increase in the glycinate group, but no changes were observed in the gluconate or placebo groups. Neither compound was found to affect copper status [208]. These results are in agreement with those previously obtained by Gandia et al., who also demonstrated the superiority of zinc bis-glycinate over zinc gluconate in terms of bioavailability [209]. In a double-blind, three-period crossover design by Piacenza et al., eight healthy subjects were given three different zinc salts: gluconate, aspartate, and sulfate, alternately ingested with 200 mL of cow’s milk. The ratio of ^66^Zn and ^70^Zn isotopes in urine was then determined before and 48 h after zinc supplementation. The highest FZA (fractional zinc absorption) value was observed for zinc aspartate (34.58%). It was also confirmed that organic forms predominated over zinc sulfate in the amounts of zinc absorbed (19.13% and 8.94% for gluconate and sulfate, respectively) [210].

In addition to amino acids and peptides, plant polysaccharides are also potential natural compounds offering zinc in a form with better bioavailability. These complexes, as shown in in vivo and in vitro studies, were characterized by better bioavailability than other zinc supplements [211]. Zinc polysaccharide compounds exhibit broad biological effects. Zinc complexes with polysaccharides from pumpkin skin (*Cucurbita moschata*) exhibited protective properties against CuSO_4_-induced inflammatory reactions in zebrafish [212]. Complexes with polysaccharides from *Athelia rolfsii*, in addition to antioxidant activity, showed greater efficacy in treating zinc deficiency than organic and inorganic forms of Zn [213]. In turn, polysaccharides from *Dictyophora indusiata* bind zinc and exhibit greater antiproliferative effects compared to ZnCl_2_ [214]. Complexes with polysaccharides derived from edible mushrooms have also gained research interest in the context of increasing zinc bioavailability. These complexes have been shown to have antioxidant, anti-inflammatory, hypoglycemic, and hepatoprotective effects [215]. Jäger et al. have shown that the glycosylation process by probiotics and yeasts has the potential to increase the bioavailability of minerals. In comparison with zinc oxide, the combinations of this element with a glycoprotein matrix were characterized by increased bioavailability [216]. Interestingly, Martinez et al. reported that a compound found in olive oil, hydroxytyrosol, increased the bioavailability of zinc from meat enriched with inorganic zinc salts [217].

The use of proton pump inhibitors may affect the absorption of minerals. When discussing the bioavailability of zinc from supplements, it is worth mentioning this group of drugs that have a potential impact on zinc solubility [218]. Farrell et al. compared zinc status and absorption between healthy individuals not taking any medications and patients with gastroesophageal reflux disease (GERD) who had been using proton pump inhibitors (PPIs) for over six months. Both groups were given zinc gluconate twice daily for 14 days, providing 26.2 mg of elemental zinc per dose. Plasma Zn levels in the PPI group increased by only 37%, compared to a 126% increase in the control group. This demonstrates that elevated gastric pH—caused by prolonged PPI use—significantly impairs Zn absorption [219]. In turn, Kaczmarczyk et al. showed that zinc levels in PPI patients were higher than in the group of healthy individuals [220]. A summary of Zn bioaccessibility/bioavailability studies using different experimental models was summarized in Table 3.

## 6. Conclusions

Zinc is a trace element that is involved in many physiological processes. Its proper supply in the diet is essential for maintaining health. The human body partially regulates its absorption in the digestive tract. However, other food components affect the absorption process of this element. The complexity of the composition of the food matrix results in mutual interactions, often difficult to define. The main compounds that hinder Zn absorption are phytates, and the role of Ca and Fe in Zn absorption is not entirely consistent. On the contrary, proteins and their hydrolysis products, i.e., peptides and amino acids, have a beneficial effect on zinc bioavailability. It is also important to consider the chemical form in which it occurs for bioavailability. Both in vitro and in vivo studies have shown higher bioaccessibility/bioavailability of Zn from organic forms compared to inorganic forms. Results are often inconclusive between in vitro and in vivo models. Zinc glycinate showed an advantage over gluconate in in vitro models and in clinical studies. On the other hand, clinical studies revealed no significant differences between the bioavailability of zinc citrate and gluconate, although in in vitro studies Zn gluconate was characterized by better bioaccessibility. Similarly, when it comes to comparing the bioavailability of Zn-methionine and Zn-glycinate, the results in animal studies do not match the in vitro studies. Animal studies have shown an advantage of the combination with methionine. Amino acid connections with zinc can be transported through membranes by amino acid transporters.

Due to the prevalence of Zn deficiency, approaches to supplementation and food fortification aimed at increasing bioavailable zinc are still being sought. Complexes with other compounds, especially polysaccharides, show potential as new forms of zinc supplements due to their improved bioavailability and broad biological effects.

A key aspect of zinc bioavailability studies is the selection of an appropriate research method. In this respect, in vitro digestion models simulating the gastrointestinal tract are commonly used. Models using Caco-2 cells seem to yield the best results. In vitro methods provide an opportunity for a preliminary estimate of zinc bioaccessibility and also allow for determining the effect of selected compounds contained in food. Human studies seem to be the most appropriate because they fully reflect all stages of Zn transport, from absorption, through distribution and metabolism, to excretion. However, these studies are very expensive, have ethical limitations, are very time-consuming, and often involve a small group of people. The results of animal studies are difficult to extrapolate to the results of bioavailability in humans. In summary, there is no ideal method for assessing Zn bioaccessibility/bioavailability. Each method has its advantages and disadvantages, and it should be noted that the results obtained using different techniques are not always complementary. It is important that the authors provide precise details of the methodology in their studies and correctly use the terms bioaccessibility/bioavailability. Despite significant improvements in this area in recent years, these terms are still used incorrectly.

In studies of zinc bioavailability, harmonization of research methodologies is crucial. For in vitro investigations, the INFOGEST protocol has been established as a standardized digestion model. However, it is essential to select methods that not only simulate in vitro digestion but also accurately replicate intestinal absorption processes. Animal studies utilize a variety of models, making it imperative to identify or develop an optimal model whose outcomes closely correlate with human physiology. Establishing standardized animal models and parameters would greatly facilitate comparability across studies. Conversely, human studies would benefit from larger and more diverse cohorts, including individuals with health conditions and those taking medications that may affect mineral absorption. A critical step forward is the implementation of randomized, long-term clinical trials assessing zinc bioavailability. It is also crucial to determine effective zinc biomarkers in humans.

## Figures and Tables

**Table 1:** Major factors of zinc deficiency [45,47,52,59,60,61].

Factor of Deficiency	Individual Factor
Improper diet	Plant-based diet Poverty—lack of access to food Malnutrition
Altered intestinal absorption	Connatural—genetic mutation of ZnT transporters (*Acrodermatitis* *enteropathica*-AE) A diet high in absorption inhibitors (phytates, lignins, fiber)
Increased loss of Zn	Diarrhea and diarrhea-related diseases Renal diseases
Interactions with drugs and trace elements	Diuretics, angiotensin-receptor blockers, proton pump inhibitors, drugs and dietary supplements rich in Ca, Fe, Se, Cu
Diseases	Chronic inflammatory disease, diabetes mellitus, alcoholism
Increased demand	Pregnancy, lactation, preterm birth, the elderly, puberty
Others	Stress, burns, surgery, infections, intravenous and enteral alimentation

**Table 2:** Comparison of benefits and limitations of in vitro and in vivo studies [12,105,113].

Method of Studies	Benefits	Limitations
In vitro studies	Simplicity	Partial reflection of the condition of the human digestive tract
	Low cost	Exclusion of oral phase (very often) Colonic phase exclusion (mostly)
	Free from ethical aspects Multiple sample analysis Determining the impact of a specific food components High repeatability	Physiological factors, age, gender, and health condition are hard to evaluate
In vivo studies		Expensive, complicated, time-consuming
	Physiological, health, gender, and age factors are considered	Limited by ethical aspects (especially in the case of assessing unsafe/toxic compounds)
		The influence of difficult-to-define and control factors
		Low repeatability Difficult to extrapolate the results of animal studies to humans

**Table 3:** A summary of Zn bioaccessibility/bioavailability from different chemical forms using various experimental models.

Model	Zn Forms	Sample/Study Group	Results	References
In vitro	Zn bis-glycinate, Zn sulfate Zn picolinate, Zn citrate, Zn methionine, Zn gluconate	Dietary supplements	Highest bioaccessibility for Zn bis-glycinate was 5.77–9.38%, lowest for Zn sulfate, 1.13% Better bioaccessibility for capsules (6.19%) than tablets (4.48%)	[116]
	Zn-amino acid complexes (Zn-Aas)/ZnCl_2_	Zn-amino acid complex	Better availability of amino acid complexes than ZnCl_2_, use of amino acid transporters by Zn-AAs	[10]
	Pentapeptide- Zn chelate from Sweet Almond Expeller Amandin/Zn-gluconate, ZnSO_4_	Experimental material	Higher solubility and zinc transport capacity of Zn-chelate than zinc gluconate and sulfate. Better absorption of zinc gluconate than sulfate.	[198]
	Zn-osteopontin complex (Zn-OPN)	Experimental material	Increased zinc absorption in the presence of phytates compared to inorganic forms	[129]
	Organic form (Spirulina) and Zn-gluconate	Spirulina tablets	Better bioaccessible Zn from Spirulina tablets than from Zn-gluconate	[184]
	Succinyled sodium caseinate Zn complex (S.NaCN-Zn)	Experimental material	Higher bioaccessibility of S.NaCN-Zn than from ZnSO_4_	[130]
	Zn contained in *Chlorella vulgaris*	Dietary supplements with *Chlorella vulgaris*	Negligible bioaccessibility of zinc	[199]
Animal studies	Zn-methionine, Zn-glycinate	Broilers	Increased bioavailability in line with Zn-methionine, Zn-glycine, ZnSO_4_	[140]
	Zn-glycinate/ZnSO_4_	Young lambs	The advantage of zinc glycinate	[200]
	Zn-threonine/ZnSO_4_	Broiler chickens	The advantage of Zn-threonine	[201]
	Basic ZnCl_2_/ZnSO_4_	Broilers	The advantage of basic ZnCl_2_	[202]
	Zn-protein complex/ZnSO_4_	Broilers	Better bioavailability from Zn-protein complex	[203]
Clinical studies	Zinc oxide/ZnSO_4_	10 women aged 21–51 years	Similar absorption of Zn from both forms	[204]
	Zinc oxide/ZnSO_4_	22 adult men	Similar absorption of Zn from both forms	[205]
	Zn citrate, Zn gluconate, Zinc oxide	15 adults (male and female) aged 18–45 years	Zn absorption from gluconate is estimated at 60.9%, from citrate 61.3%, from zinc oxide 40.9%	[21]
	Zn glycinate, Zn gluconate	30 women aged 18–24 years	Plasma Zn status increased in the glycinate group, with no changes in the gluconate and placebo groups	[208]
	Zn gluconate, Zn aspartate, ZnSO_4_	8 adults (male and female) aged 25–50 years	FZA for Zn aspartate was 34.58%, for Zn gluconate 19.13%, for sulfate 8.94%	[210]
	Zn glycinate, Zn gluconate	12 women aged 18–40 years	The superiority of Zn bis-glycinate	[209]

## Abbreviations

The following abbreviations are used in this manuscript:

AAS	Atomic Absorption Spectrometry
AE	Acrodermatitis enteropathica
ALP	Plasma Alkaline Phosphatase
AUC	The Area Under the Curve
CPP	Casein Phosphopeptide
CuZn-SOD	CuZn Superoxide Dismutase
DMT1	Divalent Metal Ion Transporter
FZA	Fractional Zinc Absorption
FAZ	Fractional Absorption of Zinc
HPIC	High-performance Ion Chromatography
ICP-MS	Inductively Coupled Plasma-Mass Spectrometry
ICP-OES	Inductively Coupled Plasma-emission coupled Spectrometry
PRI	Population Reference Intake
RDA	Recommended Dietary Allowances
SEC-ICP-MS	Size-Exclusion Chromatography Coupled to Inductively Coupled Plasma-Mass Spectrometry
TAZ	Total Absorption of Zinc
UL	Tolerable Upper Intake Level
